# A multi-centre international quality control study comparing mRNA splicing assay protocols and reporting practices from the ENIGMA consortium

**DOI:** 10.1186/1897-4287-10-S2-A87

**Published:** 2012-04-12

**Authors:** P Whiley, LC Walker, M De LA Hoya, B Wappenschmidt, A Becker, A Blanco, MJ Blok, MA Caligo, C Chatfield, F Couch, O Diez, L Fachal, L Guidugli, S Gutiérrez Enríquez, T Hansen, C Houdayer, S Imrie, A Lafferty, C Lázaro, M Menéndez, M Montagna, G Montalbán, M Santamariña, I Sokilde Pederson, M Southey, M Tancredi, A Tenès, M Thomassen, A Van Overeem Vega, AB Spurdle, MA Brown

**Affiliations:** 1 Genetic & Population Health Division, Queensland Institute of Medical Research, Brisbane, Queensland, Australia; 2 School of Chemistry and Molecular Biosciences, University of Queensland, Australia; 3 Peter MacCallum Cancer Centre, St Andrews Place, East Melbourne, Victoria, Australia; 4 Genetic Diagnosis Unit, Hereditary Cancer Program, Institut Català d’Oncologia, Barcelona, Spain; 5 Department of Clinical Genetics, Odense University Hospital, Odense C, Denmark; 6 Department of Obstetrics & Gynecology, University Hospital Cologne, Cologne, Germany; 7 Genomic Medicine, Department of Clinical Biochemistry, Rigshospitalet, Copenhagen, Denmark; 8 Laboratorio Oncología Molecular, Hospital Clínico San Carlos, Madrid, Spain; 9 Department of Clinical Genetics, Maastricht University Medical Centre, Maastrict, The Netherlands; 10 Fundación Pública Galega de Medicina Xenómica-SERGAS, Grupo de Medicina Xenómica-USC, CIBERER, IDIS, Santiago de Compostela, Spain; 11 Génétique Constitutionnelle, Institut Curie, Paris, France; 12 Instituto Oncologico Veneto, U.O.C. Immunologia e Diagnostica Molecolare Oncologica, Padua, Italy; 13 Department of Laboratory Medicine and Pathology, Mayo Clinic College of Medicine, Rochester, USA; 14 Department of Pathology, University of Melbourne, Melbourne, Victoria, Australia; 15 Oncogenetics Laboratory, Vall d’Hebron Institute of Oncology (VHIO), University Hospital Vall d’Hebron, Barcelona. Spain; 16 Istituto di Anatomia Patologica, Università di Pisa, Pisa, Italy; 17 Section of Molecular Diagnostics, Department of Clinical Biochemistry, Aalborg University Hospital, Aalborg, Denmark; 18 West of Scotland Regional Genetics Service, Glasgow, Scotland, UK

## 

Classification of intronic and predicted missense changes in the breast cancer susceptibility genes *BRCA1* and *BRCA2* remains a significant challenge for management of patients carrying these variants. Defective mRNA splicing is established as a pathway to disease, and mRNA analysis of unclassified variants has been shown to assist in classification and genetic counselling. However the interpretation of splicing assay results can be difficult, particularly for those variants that give rise to aberrations in a background of naturally occurring isoforms.

The ENIGMA (Evidence-based Network for the Interpretation of Germline Mutant Alleles) consortium was set up to facilitate research and improve research methods used to classify rare variants in the *BRCA1* and *BRCA2* (and potentially other) breast cancer predisposition genes. ENIGMA has established a Splicing Working Group, with stated purpose to pool the expertise of different active research groups to conduct large-scale studies that improve the clinical classification of likely spliceogenic variants. An initial project of the Splicing Working Group is to assess the consistency of protocols and results obtained across the multiple participating laboratories from Australia, Europe, UK and the USA. A comparison of mRNA assay protocols in use across 21 labs has identified differences in source material for RNA assays (cultured and uncultured lymphocytes, lymphoblastoid cell lines (LCLs) or constructs), differential use of nonsense-mediated decay inhibitors, and numerous differences in mRNA extraction, DNase treatment and cDNA synthesis methods. A second phase of the project is now underway to determine the impact of the splicing assay methods routinely used by these laboratories on assay data and clinical interpretation of a panel of variants. LCLs were selected from the kConFab repository from carriers of a variant associated with single major aberrant mRNA transcript absent in controls (n=4); carriers of a variant associated with a complicated aberrant mRNA splicing profile involving multiple transcripts including naturally occurring isoforms (n=5); female cancer-free controls (n=11). LCLs have already been distributed to 15 of 20 participating sites, and mRNA assays are underway. Preliminary results indicate that major aberrations associated with several variants mirror results previously observed for mRNA from uncultured lymphocytes. In addition, there is evidence for notable differences in expression of some isoforms compared to results previously observed for RNA from uncultured lymphocytes . This collaborative effort will provide information to inform optimal standardised mRNA splicing assay methodology, and to improve guidelines for clinical interpretation of assay results.

